# Effect of Ball Burnishing Pressure on Surface Roughness by Low Plasticity Burnishing Inconel 718 Pre-Turned Surface

**DOI:** 10.3390/ma15228067

**Published:** 2022-11-15

**Authors:** Pengcheng Cui, Zhanqiang Liu, Xinglin Yao, Yukui Cai

**Affiliations:** 1 School of Mechanical Engineering, Shandong University, Jinan 250061, China; 2 Key Laboratory of High Efficiency and Clean Mechanical Manufacture of MOE, Key National Demonstration Center for Experimental Mechanical Engineering Education, Jinan 250061, China; 3 Sinotruk Jinan Axle Co., Ltd., Jinan 250061, China

**Keywords:** surface roughness, low plasticity burnishing, LPB pressure, Hertz contact mechanics, slip-line field theory

## Abstract

The low plasticity burnished surface roughness is significantly affected by the low plasticity burnishing (LPB) parameters. This research proposed the analytical prediction model to predict the LPBed surface roughness and optimal LPB pressure based on Hertz contact mechanics and the slip-line field theory. In this study, the surface formatted process was divided into the smoothing stage (SS) and the indentation stage (IS). The smoothing mechanism of SS and the deterioration mechanism of IS were analyzed theoretically. The analytical prediction model for the LPBed surface roughness was proposed based on Hertz contact mechanics and slip-line field theory. The proposed analytical prediction model was validated by the LPBed surface roughness of AISI 1042, and the error of the analytically predicted results was less than 13.3%. After validation, the proposed model was applied to predict the LPBed surface roughness of Inconel 718. The single-factor experiments were conducted. The error between the proposed model prediction results and experimental results was less than 7% for the LPBed surface roughness of Inconel 718. The optimal LPB pressure interval was calculated to be [12.2 MPa, 17.5 MPa], corresponding to the experimental one as [12 MPa, 18 MPa]. It indicated that the proposed model could accurately predict the LPBed surface roughness and conduct the LPB processing.

## 1. Introduction

**Figure 1:**
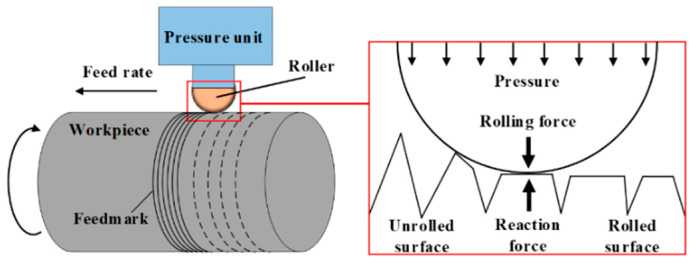
Surface smoothing mechanism by LPB process.

Low plasticity burnishing (LPB) is commonly used to lower surface roughness [1,2,3] since the rough surface weakens the fatigue properties of the material [4]. The LPB equipment generally consists of a pressure unit, a hydraulic circuit and a roller. The hydraulic pressure supplied by the pressure unit is transmitted to the roller through the hydraulic circuit and finally applied to the surface in form of contact stress. The mechanism of LPB is shown in Figure 1. The surface integrity is significantly affected by LPB pressures. The higher LPB pressure induces higher compressive residual stress [5,6]. However, the optimal interval of the LPB pressure is also limited by surface roughness.

The effect of the LPB pressure on surface roughness has been studied mainly by experiments. The LPB pressure was considered one of the most remarkable parameters for surface roughness [7,8]. The surface roughness could be optimized by increasing LPB pressures because of the more adequate plastic deformation [9,10]. However, many studies have demonstrated that surface roughness could deteriorate under excessive LPB pressures [11,12,13]. The surface roughness deterioration under larger LPB pressures was observed experimentally. However, it was seldomly considered in the surface roughness prediction models, which led to large prediction errors under larger LPB pressures.

**Figure 2:**
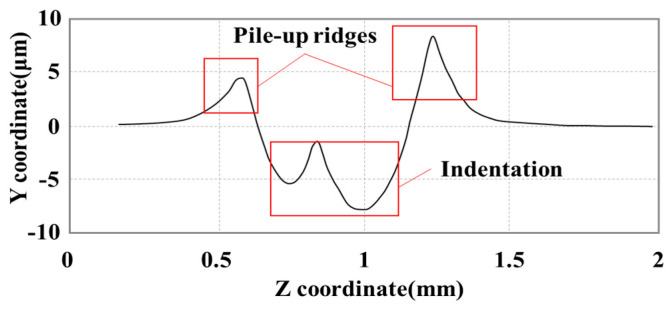
Simulated LPB surface profile with pile-up obtained. (Reprinted with permission from Ref. [16]; published by Elsevier, 2013).

The material pile-up caused by excessive pressures induced worse surface roughness. Magalhães et al. [14] found that the rolled surface roughness increased with the rolling pressure. They explained this fact by work material flow caused by the increase in deep rolling pressure. Bougharriou et al. [15] observed the deterioration of surface roughness at high rolling penetration depth by finite element simulation. The deterioration was the result of the upward escape of surface material caused by high burnishing force. Balland et al. [16] presented the LPBed surface profile by the finite element simulation. As shown in Figure 2, both indentation and pile-up ridges formatted during rolling could be observed on the LPBed surface, which significantly affected the surface roughness. The pile-up was also found in Dai’s and Öpöz’s experiments of Inconel 718 [17,18].

The experimental and analytical models have already been used to analyze the relationship between rolling pressures and surface roughness. Zhang et al. [19] proposed a prediction model of the LPBed surface roughness based on machining experiments. However, the model was compatible only under experimental conditions. The material pile-up observed by Zhang helped to analyze the surface deterioration under excessive pressure. Bouzid et al. [20] proposed an analytical surface roughness model based on Hertz contact mechanics. However, plastic deformation was not considered, which was inconsistent with the finishing mechanism of the rolling process [14]. Mieczylaw et al. [21] revealed the finishing mechanism of the rolling process. They proposed an analytical model to optimize the rolling force for better surface roughness. The burnishing force for 42CrMo4 was optimized; the model cannot be used to predict the LPBed surface roughness. Li et al. [22] proposed an analytical model for LPBed surface roughness, in which the surface roughness peaks were simplified as wedges, and elastoplastic mechanics were used to analyze the deformation of the surface material. The model was used to predict the surface roughness of AA 7050 and AISI 5140. However, the model cannot predict surface deterioration under excessive pressures or optimize the LPB pressure. The finishing effect of LPBed surface roughness could be predicted by the models in published references, in which the deterioration under high pressures was rarely analyzed. The results were thus in the inaccurate prediction of the LPBed surface roughness under excessive LPB pressures. In this study, the analytical prediction model considering the deterioration under higher LPB pressures was proposed.

**Figure 3:**
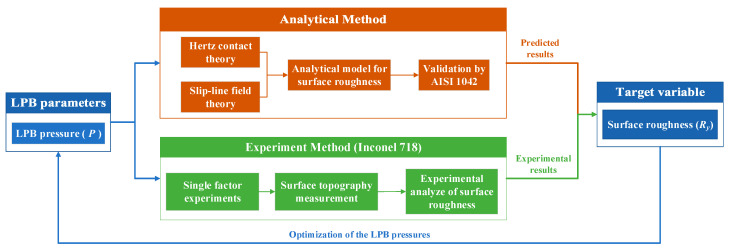
Analytical and experimental study framework.

The purpose of this research is to propose the analytical prediction model for the LPBed surface roughness. The surface deterioration under excessive pressures is considered in the proposed model. The LPBed surface roughness and the optimal LPB pressures can be predicted accurately with the proposed model. The flow diagram of this research is shown in Figure 3. Firstly, the analytical prediction model for the LPBed surface roughness was proposed based on Hertz contact mechanics and slip-line field theory. Then, the model was validated by the LPBed surface roughness of AISI 1042 from the research of Bougharriou [15,23]. Finally, the proposed model was applied to predict the LPBed surface roughness of Inconel 718. The single-factor experiments were carried out to validate the predicted results. The optimal range of the LPB pressure was obtained, which could guide the high-quality LPB processing.

## 2. Theoretical Modeling of Low Plasticity Burnished Surface Roughness

### 2.1. Surface Formation Mechanism of LPB

**Figure 4:**
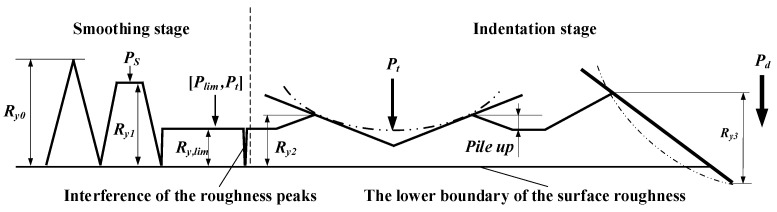
Surface morphologies after smoothing stage and indentation stage.

The LPB surface formatted process was divided into two stages including the smoothing stage (SS) and the indentation stage (IS) according to the behavior of the surface formation. The surface morphologies under different pressures were depicted in Figure 4.

The surface roughness was decreased with the increase in the LPB pressure in the smoothing stage. There were no indentations or pile-ups formed in SS because of the low LPB pressures. The deformed surface roughness peak interfered with the adjacent ones when the LPB pressure reached the limit value (*P_lim_*). It signified that the surface roughness reached the minimum value. The surface roughness was stable at the minimum value until the pressure exceeded the transforming pressure (*P_t_*), which connotes the end of SS. Therefore, the optimal LPB pressure interval was [*P_lim_*, *P_t_*], as shown in Figure 4.

The LPB process came to IS as the pressures increased beyond *P_t_*. The surface material could be extruded out and piled up near the indentation. The surface roughness was only deteriorated by the pile-up when the pressure was less than the deep indentation pressure (*P_d_*). The surface roughness was deteriorated by the combined effects of the indentation and pile-up when the pressure is higher than *P_d_*.

The analytical prediction model for the LPBed surface roughness was established based on Hertz contact mechanics and slip-line field theory. The ten-point average height *R_y_* which could accurately reflect the surface roughness was employed for the surface roughness assessment. Several assumptions of the prediction model were summarized as follows:

(a) The turned surface roughness peaks are simplified to wedge shapes.
(b) The roller is simplified as a line since the diameter of the roller is much larger than the surface roughness peak.
(c) The plastic deformation of the surface wedge is larger compared to that of the elastic deformation. Therefore, the material of the surface roughness peak is assumed as the ideal rigid-plastic material and the roller rigid body.
(d) The frictional coefficient between the ball and the workpiece is 10^−5^~5 × 10^−3^ [24]. Therefore, the surface is assumed to be frictionless to simplify the model derivation.
(e) The surface is flat when the surface roughness reaches the minimum value.
(f) The low boundary of surface roughness is not affected by the pile-up.

### 2.2. Modeling of Surface Roughness in SS

The surface roughness wedge was flattened under the contact load of the roller. The plastic deformation increased as the LPB pressure increased in this stage. That resulted in a decrease in the surface roughness with the pressure increasing. However, the minimum value could not be decreased unlimitedly. The surface roughness reached the limit value when the peaks interfered with each other.

**Figure 5:**
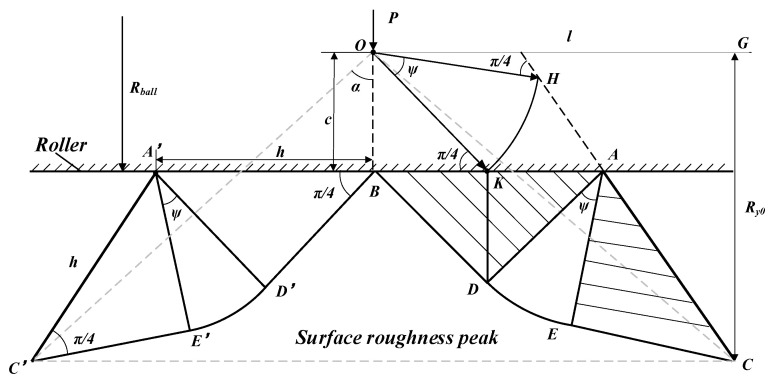
Plastic deformation of surface roughness peak in SS.

The deformation of the surface roughness peaks in SS was shown in Figure 5. The wedge top angle (*α*), the initial surface roughness (*R_y_*_0_) and the half-width of the wedge (*l*) could be measured from the tool marks. *P* is the LPB pressure. The press depth (*c*) and the half-width of the contact area (*h*) could be expressed as Equation (1). The slip-line angle (*ψ*) could be calculated by Equation (2) [22,25].

(1) $$c=\frac{h\cos\psi }{\sin\psi +1},$$

(2) $$\tan\alpha =\frac{{\left(1+\sin\psi \right)}^{2}}{\cos\psi (2+\sin\psi )},$$

According to the slip-line field theory, the force of the rolling ball is balanced and the surface roughness peak deformation is terminated when the rolling force satisfies Equation (3).

(3) $$\frac{F}{2h}=2k(1+\psi ),$$

where *h* is the half-width of the contact area. *k* is the parameter related to the material yield criterion. For the von Mises yield criterion, $k=\sigma_{s}/\sqrt{3}$, where *σ_s_* is the yield strength of workpiece material. The rolling force *F* can be calculated by Equation (4) for the LPB process [26].

(4) $$F=P\pi R_{ball}^{2},$$

The predicted surface roughness in the smoothing stage can be expressed as Equation (5).

(5) $$R_{y}=R_{y0}-c,$$

where *R_y*0*_* is the initial surface roughness.

The deformed peak interfered with the adjacent ones when the contact length (*h*) equals the half-width of the wedge (*l*). According to Equations (1) to (5), the limit pressure (*P_lim_*) corresponding to the minimum surface roughness could be expressed as Equation (6).

(6) $$P_{\lim}=\frac{4\sigma_{s}(1+\psi )}{\sqrt{3}\pi R_{ball}^{2}}l,$$

Combining Equations (1) to (5), the surface roughness in SS could be expressed as Equation (7) based on the Hertz contact mechanics and slip-line field theory.

(7) $$R_{y}=\left\{ \begin{array}{l} R_{y0}-\frac{\sqrt{3}P\pi R_{ball}{}^{2}\cos\psi }{4\sigma_{s}(1+\psi )(\sin\psi +1)},P<P_{\lim}\\ R_{y0}-\frac{R_{y0}\tan\alpha \cos\psi }{1+\sin\psi },P\geq P_{\lim} \end{array},\right.$$

where the yield strength of the material (*σ_s_*) and the radius of the roller (*R_ball_*) are the constants of the workpiece and the roller. The only input variable is the LPB pressure (*P*).

### 2.3. Modeling of Surface Roughness in IS

Surface roughness tended to be worse when the LPB pressure exceeded *P_t_*. That was because the residual indentation occurred under the excessive LPB pressures. The extruded material piled up surrounding the indentation, and the surface roughness was aggravated. The slip-line field theory was used to calculate the height of the pile-up. By superimposing it with the *R*_lim_, the surface roughness of the IS could be finally obtained [25].

**Figure 6:**
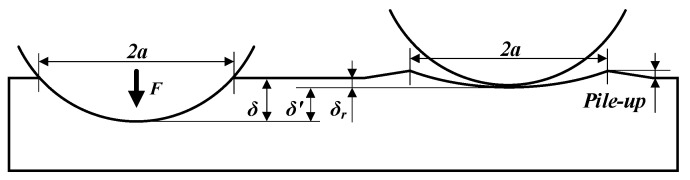
Surface profile of IS during loading and unloading.

The formation process of the pile-up is shown in Figure 6. The workpiece was simplified as a plane and the roller had a rigid body [27]. *a* shown in Figure 6 is the radius of the contact area. The total displacement (*δ*) including the elastic and plastic deformation were both induced when the pressure loaded. The elastic displacement (*δ′*) recovered when the pressure was unloaded, and the plastic displacement (*δ_r_*) remained on the surface. The material surrounding the indentation was higher than the initial surface [28]. The pile-up was only related to the residual indentation. Therefore, *δ_r_* needs to be calculated first, which is shown in Equation (8).

(8) $$\delta_{r}=\left\{ \begin{array}{l} \delta -\delta^{\prime },\delta >\delta^{\prime }\\ 0,\delta \leq \delta^{\prime } \end{array}\right.,$$

The total displacement *δ* could be calculated with Equation (9). The LPB force (*F*) could be calculated with Equation (4). *R* and E* are the equivalent radius and the equivalent elasticity modulus, which could be calculated with Equation (10). *R*_1_, and *R*_2_ are the radius of the rolling ball and the workpiece to be machined, respectively*. E*_1_, *E*_2_, *ν*_1_, *ν*_2_ are the elastic modulus and Poisson’s ratio of the roller and the workpiece material, respectively [29].

(9) $$\delta =\sqrt[{\begin{array}{l} 3 \end{array}}]{\frac{9F^{2}}{16RE^{*2}}},$$

(10) $$\left\{ \begin{array}{l} \frac{1}{R}=\frac{1}{R_{1}}+\frac{1}{R_{2}}\\ \frac{1}{E^{*}}=\frac{1-\nu_{1}^{2}}{E_{1}}+\frac{1-\nu_{2}^{2}}{E_{2}} \end{array}\right.,$$

The elastic displacement (*δ′*) could be calculated by Equation (11). The *F*_Y_ and *δ*_Y_ shown in Equation (11) are the critical load and displacement corresponding to the beginning of the surface plastic deformation, which could be calculated with Equations (12) and (13), respectively. *P*_0Y_ is the maximum contact stress when the surface material yields. *P*_0Y_ = 1.6*σ_s_* for the von Mises yielding criterion [29].

(11) $$\frac{F}{F_{Y}}=0.38{\left(\frac{\delta^{\prime }}{\delta_{Y}}\right)}^{2},$$

(12) $$F_{Y}=\frac{\pi^{3}R^{2}}{6E^{*}{}^{2}}{\left(P_{0Y}\right)}^{3},$$

(13) $$\delta_{Y}=\sqrt[{\begin{array}{l} 3 \end{array}}]{\frac{9F_{Y}{}^{2}}{16RE^{*2}}},$$

The residual indentation *δ_r_* could be calculated with Equations (8) to (13). The surface formation stage went into SS when *δ_r_* > 0. The transforming pressure (*P_t_*) corresponding to the beginning of the SS could be expressed as Equation (14) by substituting *δ′* = *δ* into Equation (11).

(14) $$P_{t}=\frac{0.38F_{Y}}{\pi R_{ball}^{2}}{\left(\frac{\delta }{\delta_{Y}}\right)}^{2},$$

The material pile-up formed on the surface and the roughness deteriorated when the pressure exceeded *P_t_*. Therefore, the optimal interval of the LPB pressure could be expressed as [*P*_lim_, *P_t_*].

**Figure 7:**
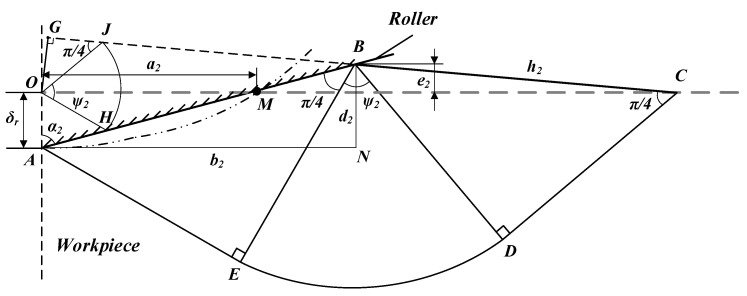
Slip-line field distribution and surface material deformation during IS stage.

The pile-up was analyzed by slip-line field theory, which was shown in Figure 7. The plastic deformation of the curved indentation was far more complicated than the linear one. The indentation was simplified to the wedge referring to the idea of Black [30]. *AEDC* was the velocity interruption line, and *OJH* was the velocity end diagram. *δ_r_* was the residual displacement calculated above. According to the geometrical considerations, the width of residual indentation (*a*_2_) could be expressed as Equation (15).

(15) $$a_{2}=\sqrt{2R_{ball}\delta_{r}-R_{ball}^{2}},$$

The simplified wedge angle (*α*_2_) could be obtained with Equation (16).

(16) $$\alpha_{2}=\arctan\left(\frac{a_{2}}{\delta_{r}}\right),$$

The relationship between *α*_2_ and the slip-line angle (*ψ*_2_) is shown in Equation (17).

(17) $$\cos(2\alpha_{2}-\psi_{2})=\frac{\cos\psi_{2}}{1+\sin\psi_{2}},$$

By the geometric relationship shown in Figure 7, the contact length (*h*_2_) could be calculated with Equation (18).

(18) $$h_{2}=\frac{\delta_{r}}{\cos\alpha_{2}-\sin(\alpha_{2}-\psi_{2})},$$

The height of the peak-valley (*d*_2_) could be expressed in Equation (19).

(19) $$d_{2}=\frac{\delta_{r}\cos\alpha_{2}}{\cos\alpha_{2}-\sin(\alpha_{2}-\psi_{2})},$$

The pile-up height (*e*_2_) could be obtained by Equation (20).

(20) $$e_{2}=\delta_{r}\left(\frac{\cos\alpha_{2}}{\cos\alpha_{2}-\sin(\alpha_{2}-\psi_{2})}-1\right),$$

The final surface roughness (*R_y_*) was only affected by *e*_2_ when *δ_r_* ≤ *R_y_*_,lim_. When *δ_r_* > *R_y_*_,lim_*,* the residual indentation depth was lower than the original boundary, and the surface roughness was affected by both *δ_r_* and *e*_2_. The *R_y_* could be expressed as Equation (21).

(21) $$R_{y}=\left\{ \begin{array}{l} R_{y0}-\frac{\sqrt{3}P\pi R_{ball}^{2}\cos\psi }{4\sigma_{s}(1+\psi )(\sin\psi +1)},P<P_{\lim}\\ R_{y0}-\frac{R_{y0}\tan\alpha \cos\psi }{1+\sin\psi },P_{\lim}\leq P<P_{t}\\ R_{y0}-\frac{R_{y0}\tan\alpha \cos\psi }{1+\sin\psi }+e_{2},P_{t}\leq P<P_{d}\\ d_{2},P\geq P_{d} \end{array}\right.,$$

### 2.4. Validation of Proposed Model Prediction Results

The proposed analytical prediction model was validated by the LPBed surface roughness of AISI 1042. The surface roughness data after the LPB process of AISI 1042 were acquired from the research of Bougharriou [15,23]. The mechanical properties and the initial surface geometric parameters of AISI 1042 were shown in Table 1.

**Figure 8:**
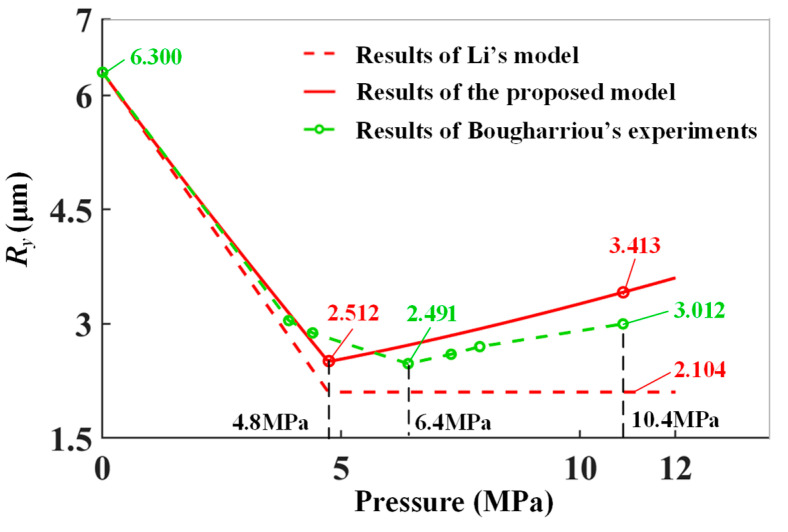
Comparison between experimental results and prediction values.

The new proposed model and the model of Li were both utilized to predict surface roughness. The optimal pressure interval (*I_OP_*) and the error of it (*E_I_*), error of the minimum surface roughness (*E_Rmin_*) and surface roughness at 10.4 MPa (*E_R10.4_*) were employed to evaluate the accuracy of the model. The experimental results and the predicted results were shown in Figure 8.

As shown in Figure 8, the surface roughness decreased to an inflection point and then deteriorated as LPB pressure increased. The experimental trend fitted well with the model prediction.

For experiments, the minimum surface roughness was 2.491 μm at 6.4 MPa. The surface roughness increased to 3.012 μm with 10.9 MPa. Bougharriou pointed out that the deterioration of surface roughness was caused by the material escaping upward. The surface material squeezed out and piled up resulting in surface deterioration.

The surface roughness was predicted by the model of Li first. The surface roughness decreased and was stable at a minimum value with LPB pressure increasing, which deviated from the experimental trend. The minimum predicted surface roughness was 2.104 μm, and the *E_Rmin_* was 15.5%. However, the predicted value at 10.9 MPa was still 2.104 μm with the *E_R_*_10.4_ of 30.1%. The predicted *I_op_* was [4.8 MPa, +∞) with the error *E_I_* = +∞, which was apparently incorrect. The predicted results indicated that the model of Li cannot accurately predict the surface roughness and optimize the LPB pressure. This is due to the pile-up under higher LPB pressures not being considered in the model of Li.

In comparison, the proposed model fitted better with the experimental results by considering the deterioration under higher LPB pressures. The minimum calculated surface roughness was 2.512 μm, and the *E_Rmin_* was 0.8%. The predicted result at 10.9 MPa was 3.413 μm, and the *E_R_*_10.4_ was 13.3%. The predicted *I_op_* was 4.8 MPa, and the *E_I_* was 25%. The proposed model could predict the surface roughness accurately compared with the model of Li, and it could be used to optimize the LPB pressure. The difference between the experiment and prediction results is due to the measurement error in the geometric parameters of tool marks. The random error of roughness peak geometric parameters could be reduced by measuring more surface roughness peaks. By means of that, the prediction accuracy of the proposed model could be improved.

## 3. Experiments

### 3.1. Materials

The workpiece material of Inconel 718 was treated with a solution and aging treatment. The material was heated to 960 °C and held for 1 h, and then air cooled to room temperature. Subsequently, the material was held at 720 °C for 8 h, then cooled to 620 °C at a speed of 50 °C/h and held for 8 h in a furnace, and finally, air-cooled to room temperature. All specimens were prepared from the same batch of material to avoid errors due to the difference in material batches. The mechanical properties of Inconel 718 and the roller are shown in Table 2 [31].

### 3.2. Experimental Method

**Figure 9:**
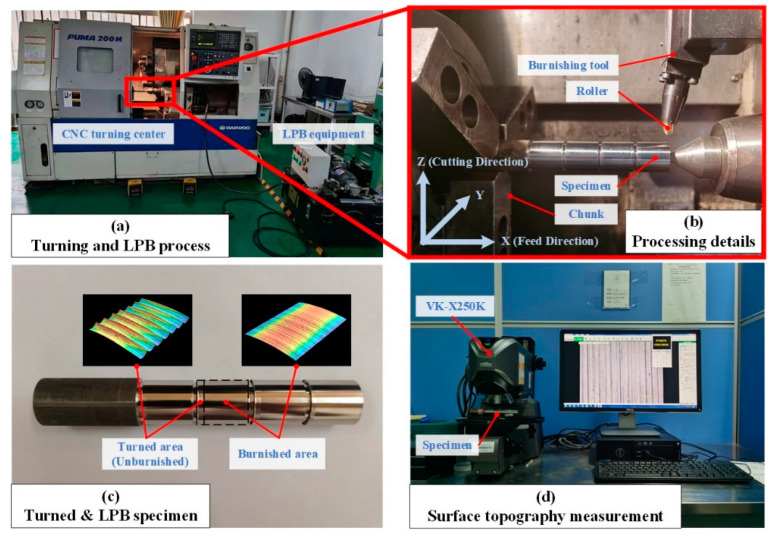
Experimental steps. (**a**) Turning and LPB process and equipment; (**b**) Processing details and the machining coordinate system establishment; (**c**) The specimens after turning and LPB process; (**d**) Surface topography instruments and measurement process.

Figure 9 depicted the experimental equipment and process. The turning and LPB processes were both carried out at the PUMA 200 MA CNC turning center (DAEWOO, Incheon, Korea). The insert used in the turning process was VBMT160404-MF1105 (Sandvik, Sandviken, Sweden). The turning processing parameters were determined as follows: turning speed *v* = 50 m/min, feed rate *f* = 0.1 mm/r, and the depth of cut *a_p_* = 0.1 mm. The size of the specimen was *ϕ*24 × 140 mm. Four rolled sections were set for each specimen to be processed with different rolling parameters.

The specimen was subjected to the LPB process after the turning process, as shown in Figure 9b. The hydraulic unit can produce a maximum pressure of 21 MPa. The diameter of the rolling ball was 6 mm. Five groups of the LPB pressures were set as 9 MPa, 12 MPa, 15 MPa, 18 MPa and 21 MPa. Two passes and 0.1 mm/r feed rate were determined according to the pre-experiments. The right-angle coordinate system was established to facilitate the description of the machining process and analysis of the data.

The surface topography and the roughness were measured by VK-X250K (Keyence, Osaka, Japan). The roughness was measured along the feed direction since it has the most significant effect on the surface property. The ten-point average height *R_y_* was selected as the surface roughness assessment index. To ensure reliability, *R_y_* for each group was measured three times and their average value was taken as the final value.

## 4. Results and Discussion

### 4.1. Experimental Results

**Figure 10:**
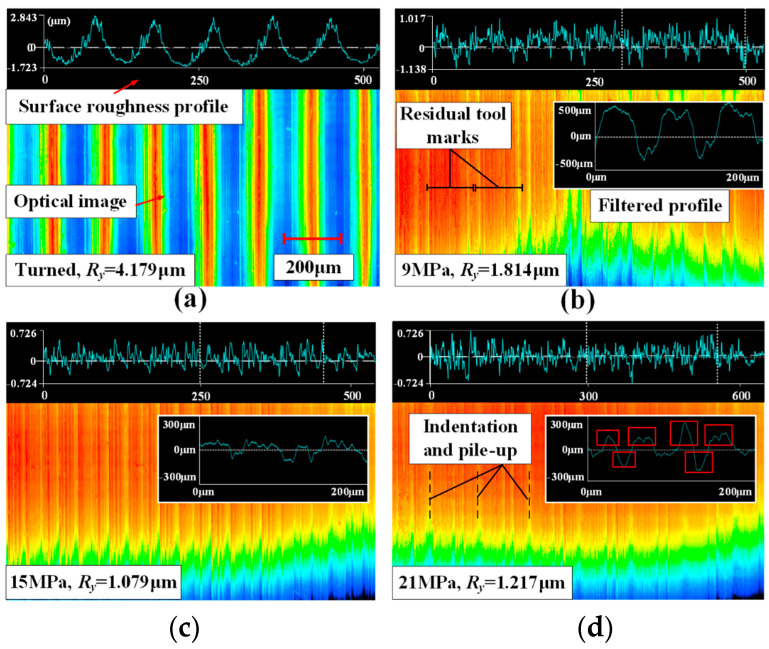
Surface topography of machined Inconel 718. (**a**) Turned, (**b**–**d**) LPBed under different pressures of 9 MPa, 15 MPa, 21 MPa, respectively.

The surface profile after turning and the LPB process was shown in Figure 10. Figure 10a depicted the tool marks on the turned surface. *R_y_* was measured to be 4.179 μm (standard deviation 0.037 μm). The periodically distributed tool marks were observed on a turned surface. The wedge shape of the tool marks supported the simplification of the model.

The tool marks tended to be smooth under the contact load in SS as shown in Figure 10b. The surface roughness decreased to 1.814 μm (s.d. 0.020 μm). However, the residual tool marks could still be observed on the surface. It was because the tool marks cannot deform sufficiently under the low LPB pressure. No interference between residual tool marks was observed, which indicates that the surface roughness could be further decreased by increasing the LPB pressure.

The surface roughness reached the minimum value when the LPB pressure came to 15 MPa. Figure 10c depicted the surface profile after the LPB process of 15 MPa. No residual tool marks or indentations were observed on the LPBed surface. However, the surface texture cannot be eliminated completely due to the interference of the peaks [22]. It indicated that the minimum surface roughness was limited. The minimum surface roughness was 1.079 μm (s.d. 0.041 μm) under the experimental conditions. Compared with the initial surface, the reduction in surface roughness was 74.2%.

The surface topography under 21 MPa was obviously worse than the one under 15 MPa. The surface roughness increased to 1.217 μm (s.d. 0.006 μm), which was 12.8% higher compared with the 15 MPa. The surface profile of the 21 MPa was apparently rougher compared with the one under 15 MPa. The indentations and pile-up were observed on the LPBed surface as shown in Figure 10d. That proved the surface deterioration resulted from the combined effects of pile-up and indentations.

### 4.2. Surface Roughness Prediction and the LPB Pressure Optimization for LPBed Inconel 718

**Figure 11:**
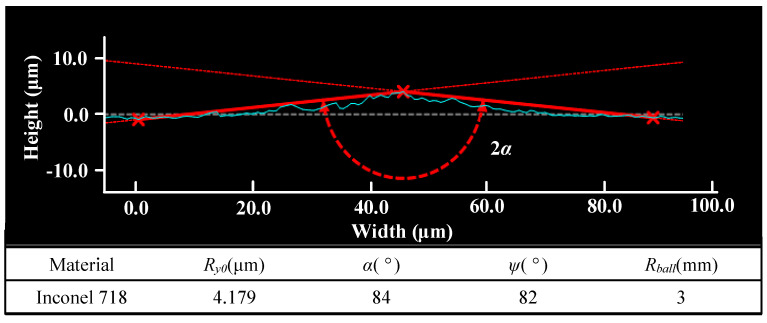
Surface roughness peak simplification and geometric parameters measurement. (The blue line is the measured surface roughness peak. The red line is the simplified wedge. The red dot line indicate the wedge angle).

The proposed model was used to predict the surface roughness under different LPB pressures. The optimal LPB pressure interval was also calculated in order to conduct the LPB process. Firstly, the geometric parameters of the initial surface were measured. The surface roughness peaks were simplified as a wedge as shown in Figure 11, and the geometric parameters were measured. Subsequently, the geometric parameters and material property parameters were subscribed to in the proposed model. Finally, the model-predicted results were compared with the experimental results. The optimal interval of the pressure (*I_OP_*), the maximum error (*E_max_*), the error of the minimum roughness (*E_Rmin_*) and the surface roughness at 21 MPa (*E_R,_*_21_) were used to evaluate the model.

**Figure 12:**
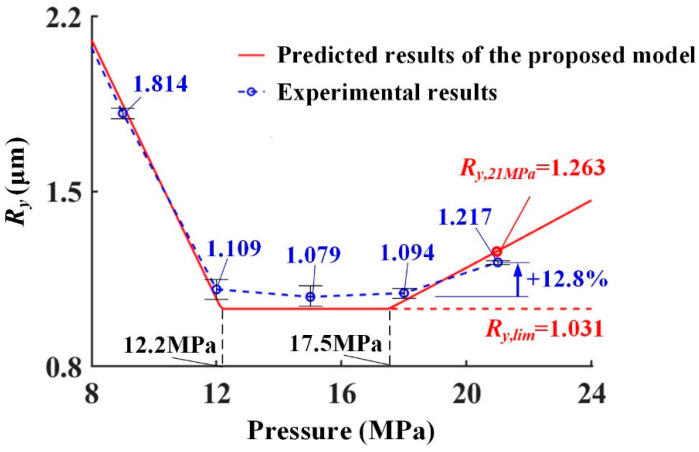
Experimental results and predicted results for LPBed Inconel 718.

The predicted results of the LPBed surface roughness were shown in Figure 12 and Table 3. The variation in the predicted surface roughness consists of the experimental results. The error of the predicted results was less than 7.0%. The minimum surface roughness (*R_y,min_*) calculated by the model was 1.031 μm with an error *E_Rmin_* 4.4%. The experimental surface roughness at 21 MPa was 1.217 μm. The predicted surface roughness under 21 MPa was 1.263 μm. The error *E_R,21_* was only 3.8%, which was much lower than the error of Li’s model (15.3%). The above evaluation proved that the proposed model could be used to predict surface roughness with high accuracy even under excessive pressures.

According to the experiments, the LPB pressure range corresponding to the minimum surface roughness (1.079 ± 0.040 μm) was from 12 MPa to 18 MPa. The interval calculated by the model was [12.2 MPa, 17.5 MPa]. The error of the predicted interval boundary was less than 2.8%. That indicated that the optimal interval of the LPB pressures could be accurately predicted by the proposed model.

## 5. Conclusions

In this study, the surface formation mechanism of the LPB process was analyzed. The analytical prediction model for the LPBed surface roughness has been proposed. The pile-up formatted under high LPB pressures was analyzed, which was first studied in this study. The LPB surface formatted process was divided into two stages including the smoothing stage (SS) and the indentation stage (IS). The analytical prediction model for the LPBed surface roughness was validated by the machining experiment of AISI 1042. Then, the LPBed surface roughness of Inconel 718 was predicted by the validated and proposed model. The single-factor experiments were designed and carried out. It was concluded that the LPBed surface roughness could be predicted precisely by the proposed model considering the pile-up. The conclusions are emphasized as follows:

(1) The analytical prediction model for the LPBed surface roughness was proposed based on Hertz contact mechanics and slip-line field theory. The increment of the surface roughness in IS was attributed to the pile-up. Considering the deterioration, the proposed model could successfully predict surface roughness under excessive pressures.
(2) The LPBed surface roughness of AISI 1042 was used to validate the proposed model. The predicted result of the minimum roughness was 2.512 μm (the error was 0.8%). Moreover, the surface roughness at 10.4 MPa was 3.413 μm (13.3%). According to the proposed model, the optimal LPB pressure corresponding to the minimum surface roughness was 4.8 MPa (25%). The results of Li’s model were 2.104 μm (15.5%), 2.104 μm (30.1%) and [4.8 MPa, +∞) (+∞%), respectively. Considering the pile-up, the proposed model could predict the surface roughness and the optimal pressures more accurately compared with the model of Li.
(3) The Inconel 718 was manufactured under different LPB pressures. The tool marks could be smoothed out by the LPB process. The minimum value of surface roughness was 1.079 μm. The surface roughness decreased by 72.9% compared with the turned surface roughness. However, the minimum surface roughness was limited. The surface roughness increased by 12.8% when the pressure reached 21 MPa. Pile-up and indentations observed under excessive pressures supported the deterioration of the surface roughness.
(4) The proposed model was used to predict the surface roughness and the optimal interval of the LPB pressure. The error in the proposed results was less than 7%. The proposed model was more accurate than Li’s model (15.3%). The predicted optimal interval (12.2 MPa to 17.5 MPa) was consistent with the experimental one (12 MPa to 18 MPa). The proposed model could be used to predict the LPBed surface roughness of Inconel 718, and further conduct the LPB process.

## Figures and Tables

**Table 1:** Mechanical properties and geometric parameters of AISI 1042.

Material	*E* (GPa)	*ν*	*σ_s_* (MPa)	*σ_b_* (MPa)	*R_y*0*_* (μm)	α (°)	*R_ball_* (mm)
AISI 1042	210	0.3	300	570	6.3	170.8	9

**Table 2:** Mechanical properties of Inconel 718 and burnishing roller.

Material	*E* (GPa)	*ν*	*σ_s_* (MPa)	*σ_b_* (MPa)
Inconel 718	205	0.3	1360.5	1502
SiN (Roller)	300	0.27	-	-

**Table 3:** Prediction results of LPBed Inconel 718 and accuracy evaluation.

Results	*R_y,min_* (μm)	*E_Rmin_*	*R_y,_*_21_ (μm)	*E_R_*_21_	*E_max_*	*I_OP_* (MPa)
Prediction	1.031	4.4%	1.263	3.8%	7.0%	[12.2, 17.5]
Experiment	1.079	-	1.217	-		[12, 18]

## Data Availability

Not applicable.
